# Impact of Clinical Pharmacist Intervention on Clinical Outcomes in the Critical Care Unit, Taif City, Saudi Arabia: A Retrospective Study

**DOI:** 10.3390/pharmacy10050108

**Published:** 2022-08-31

**Authors:** Abdullah Althomali, Ahmed Altowairqi, Afnan Alghamdi, Musim Alotaibi, Abdulrahman Althubaiti, Abdulaziz Alqurashi, Adnan Al Harbi, Majed Ahmed Algarni, Abdul Haseeb, Mohamed Hassan Elnaem, Faisal Alsenani, Mahmoud E. Elrggal

**Affiliations:** 1 Ministry of Health, King Faisal Medical Complex, Taif 26514, Saudi Arabia; 2 College of Pharmacy, Umm Al Qura University, Makkah 21955, Saudi Arabia; 3 Clinical Pharmacy Department, College of Pharmacy, Taif University, Taif 21944, Saudi Arabia; 4 Department of Pharmacy Practice, Faculty of Pharmacy, International Islamic University, Kuantan 53100, Pahang, Malaysia

**Keywords:** clinical pharmacist, clinical outcome, critical care unit, Saudi Arabia

## Abstract

(1) Objectives: Clinical pharmacists are now playing a significant role in hospitals aiming to reduce medication errors, adverse drug reactions, and healthcare costs. Therefore, the main objective of this study was to assess the interventions provided by a clinical pharmacist in the intensive care unit at the King Faisal Hospital in Taif city. (2) Methods: For this single-center retrospective study, patients were included from December 2021 to May 2022. In the present study, all the interventions of clinical pharmacists made over six months were included. The Intensive care unit (ICU) ward was covered by three clinical pharmacists, and the interventions made were categorized into four groups: (1) interventions related to indications; (2) interventions regarding safety; (3) interventions regarding dosing, and (4) miscellaneous. Descriptive statistics was applied to evaluate the results in the form of frequencies and percentages. Analysis was performed using the statistical package SPSS 20.0. (3) Results: Overall, a total of 404 interventions were recommended for 165 patients during the six- month period of study. Among them, 370 interventions (91.5%) were accepted by physicians. Among all the interventions, the majority were suggested regarding ‘indication’ (45.7%), including the addition of drugs, drugs with no indications, and duplication. The acceptance rate of clinical pharmacist intervention was 98.5%. (4) Conclusions: This retrospective study shows that clinical pharmacists played a critical role in optimizing drug therapy which could subsequently help to prevent drug-related issues and lower drug costs. More research is needed to do a thorough cost-benefit analysis.

## 1. Introduction

Every healthcare practitioner bears responsibility for patient safety. In recent years, the health care system has moved from a fee-for-service to a value-based care model, emphasizing quality of care for patients [1]. Throughout this evolution, a focus on interprofessional team-based care has emerged, with clinical pharmacists increasingly integrated as essential members of the healthcare team in various clinical disciplines [2].

Clinical pharmacy services began in the United Kingdom and the United States of America at the end of the 1960s [3]. While pharmacists have traditionally been considered to be ‘behind the glass’ dispensing medicines, they are increasingly considered critical members of the multidisciplinary care team handling complicated patient requirements [4]. The proportion of clinical pharmacist interventions accepted by physicians is also increasing for individualized dosing in multiple clinical conditions [5,6,7]. Overall, with pharmacist-provided drug protocol management, substantial reductions were noted in the length of stay, the total cost of care, drug costs, laboratory costs, and complications. [8]. Clinical pharmacists are now playing a significant role in hospitals, aiming to reduce medication errors and adverse drug reactions [9,10]. Licensed pharmacists with extensive education and training have the clinical competencies required to practice in team-based, direct patient care settings. Accredited residency training or similar post-licensure experience is mandatory for participation in direct patient care practice. Board certification is also required if the clinical pharmacist passes the qualifying standards of the Board of Pharmacy Specialties (BPS) [11]. Many peer-reviewed articles recommend clinical pharmacist participation to support team-based care, improve access and cooperation, and improve treatment quality and safety [12]. Due to the complexity of multiple routes of administration, clinical pharmacists play a major role in handling medication for critical diseases, preventing and correcting medication errors, optimizing medication therapy, and participating in professional activities that promote effective implementation in the hospital ward [13,14,15].

Medication-related problems are common in hospital wards, which occur during prescribing, transcribing, dispensing, administering, adherence to, or monitoring of a drug [11]. As the worldwide population ages, the economic burden due to medication-related problems is becoming the greatest challenge for healthcare organizations and has substantial impact on the mortality and morbidity of patients [12]. An increased number of medications results in the rising cost of newer pharmacotherapies. According to Frank R. Ernst, in the United States, the annual cost of medication-related mortality and morbidity of USD 76.6 billion resulted from medication-related problems [13]. The interventions of clinical pharmacists can effectively prevent these errors and the subsequent economic burden [14,15,16,17,18]. The core of pharmaceutical care services is to ensure the safe ordering and dispensing of the correct medication to patients receive the required information to safely and effectively use the medication [19,20,21,22,23]. In the 154 single-site studies involving the collaborative drug therapy management of pharmacists, 85% showed beneficial outcomes on patient care [24].

The lack of sufficient published data to describe the interventions of clinical pharmacists in Saudi Arabia was the rationale for conducting this study. Therefore, the main objective of this study was to assess the effect of the clinical interventions provided by clinical pharmacists related to dosage regimen and cost in the intensive care unit at the King Faisal Hospital, Taif city.

## 2. Materials and Methods

### 2.1. Study Design and Patient Selection

For this single-center retrospective study, patients were included from December 2021 to May 2022. A total of 165 patients with 404 interventions were admitted to the ICU at the King Faisal Hospital in Taif, Saudi Arabia, during the study period. This study was approved by the Ethics Committee of Taif Health Affairs in the Taif region with approval number (638) on 2 December 2021; a copy of the initial approval is attached to the supplement. This is one of the few studies that describes the clinical pharmacist activities in the ICU in a Taif healthcare setting. All clinical pharmacists working in the ICU had a basic pharmacy degree (PharmD) with clinical experience of a minimum of 3 years in ICU settings.

### 2.2. Data Collection

Data over six months (September 2021–February 2022) from the intensive care unit (ICU) at the King Faisal Complex Hospital was collected. The hospital has 800 beds, and out of them, 24 beds are for ICU. The emergency department first admits patients and then transfers them to the intensive care unit. The ICU team consists of a specialized consultant (head of the team), one resident doctor, and one clinical pharmacist. The ICU clinical pharmacists in the King Faisal Complex Hospital review patient profiles, patient progress notes, and all relevant physician orders such as lab results of the patients during the rounds with the ICU team. During the rounds, the clinical pharmacists deal with issues such as recommending drug therapy and responding to drug information requests. Clinical pharmacists record these interventions/recommendations on intervention monitoring forms and record the outcome of the interventions (acceptance or rejection by the team members).

### 2.3. Inclusion and Exclusion Criteria

All adult patients admitted in the ICU between September 2021 and February 2022 were included. Any patient aged less than 17 years and patients admitted through specializations other than the ICU team were excluded.

### 2.4. Data Management and Analysis

**Figure 1:**
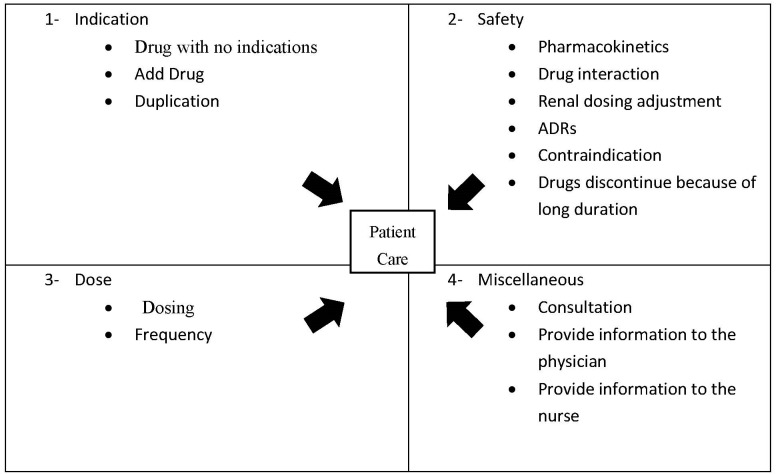
Theoretical Framework of Indicators.

The ICU ward was covered by three clinical pharmacists, and the interventions made were categorized into four groups: (1) interventions related to indications; (2) interventions regarding safety; (3) interventions regarding dosing, and (4) miscellaneous (Figure 1). Statistical techniques were applied to the data to understand the extent of the impact of the above said interventions of the clinical pharmacists on the process of patient care. Descriptive statistics was applied to evaluate the results in the form of frequencies and percentages. Analysis was performed using the statistical package SPSS 20.0.

## 3. Results

Overall, a total of 404 interventions were recommended for 165 patients during the six-month period of study. Among them, 370 (91.5%) interventions were accepted by the physicians. Among all the interventions, the majority were suggested about medication indications (45.7%), including the addition of drugs, drugs with no indications, and duplication. The addition of drugs had more frequency of occurrence (162, 79%), followed by drugs with no indications (38, 18.5%,), and duplication (five, 2.4%). Another clinical intervention involving safety accounted for 38% in second place along with therapeutic drug monitoring, dosing adjustment, the discontinuation of drugs due to long duration, and drug–drug interaction. The total number of interventions according to the indications are presented in Table 1. The interventions regarding safety revealed that among the total number of safety interventions (133): renal dose adjustment-related were 50 (37.6 %), the interventions related to drug discontinuation due to duration were 74 (55.6 %), therapeutic drug monitoring-related were seven (5.2%), and drug interaction related were two (1.50%). Interventions regarding dosing showed that the percentage of intervention regarding dosing was 85 (n = 17) and regarding frequency was 15 percent (n = 3) out of the total of 20 dosing interventions. From miscellaneous interventions, interventions regarding consultation were 30 (65%), and information for nurse-related was 16 (35%). Renal dose adjustment, drug discontinuation due to long duration, and drugs with no indications represented the most cost-saving interventions. The acceptance rate of clinical pharmacist intervention was 98.5%.

## 4. Discussion

Clinical pharmacy services have been found to improve in-hospital medication safety and patient outcomes in a significant number of cases [24]. A key aspect of hospital clinical pharmacy activity is the review of medication sheets and pharmacist interventions. Proving the economic and clinical impact of this activity appears to be necessary for a setting with limited financial resources [14,15,16]. This study assessed the impact of the review of medication sheets and pharmacist interventions, both clinically and economically. Several studies have shown that a clinical pharmacist (C.P.) plays an important role in the intensive care unit as a full multidisciplinary team member [25,26]. The full membership of a C.P. in the ICU team involves attending daily rounds in the ICU, cooperating, and being available for consultations throughout the day [27]. In ICUs, C.P.s are focused on making the best use of medication to promote patient safety and survival. Due to the critical condition of patients, polypharmacy, and rapid and frequent pharmacotherapy adjustments, the competencies of C.P.s in critical care settings are crucial. Correct drug administration, and identifying and preventing side effects, drug interactions, and pharmacotherapy errors are just a few of the essential aims for C.P.s [28]. Alternative therapy, cost-effective medicine utilization, and ICU staff education, particularly in the use of antibiotics, are all things that C.P.s should consider. It is unsurprising that the number of C.P.s has globally expanded in recent decades and that their position in many healthcare teams has become clearly defined [29,30].

These interventions are categorized into indications, safety, dosing, and miscellaneous. In our study, approximately half of all interventions were regarding the ‘indication’ (45.7%). This demonstrates the role of the clinical pharmacist in minimizing drug-related problems, which helps to improve patient safety. However, the most frequent indication intervention was regarding added drugs representing seventy-five percent. According to Grimes et al., 65.5% of all participants with medication errors have been documented at time of discharge [31]. The most common types of medication errors found in this study were inappropriate dosing, inappropriate scheduling, and drug–drug interaction at discharge. According to KK Scarsi, the pharmacist should perform activities including reviewing the prescription orders for accurate dose and medication selection to increase the safety of the patients [32]. The probability of drug–drug interaction increased with the number of drugs dispensed to patients. In this study, we recorded that about four to five drugs were dispensed to patients per prescription. Fewer drugs can decrease the chance of adverse drug effects that occur due to the interaction of drugs and can help to improve the therapeutic outcomes of patients [33]. Renal dose adjustment interventions in the safety type proposed are 50 (37.6%). So, clinical pharmacist interventions reduce the rate of drug-related problems and, as a consequence, also reduce ADRs. Thus, it is found that the participation of integrated clinical pharmacists could play an essential role in the detection and management of specialist cases such as acute or chronic kidney disease.

The clinical pharmacy services provided by pharmacists in our study have the potential to reduce the cost of pharmacotherapy. The estimation is based on the discontinuation of drugs due to: long duration, duplication, no clear indication, renal dosing adjustment, and contraindicated drugs. This is similar to another study with the same criteria that found clinical pharmacy interventions impacted the cost saving by 40% [18]. Another indicator for evaluating the importance of the clinical pharmacist is the rate of acceptance by physicians of interventions. In this study, 98.5% of the interventions are accepted by the physicians. In another study, about 98% of pharmacist intervention were accepted and implemented by the team. The degree of severity was classified as fatal, serious and significant with regards to these medication errors [34]. Integrated pharmacists play a pivotal role as medication experts to improve the safety of patients [35]. Therefore, our study suggests that pharmacists should be involved in the medication reconciliation process to assess the prescription order. They have the greatest impact on providing safe and sound medication treatment to improve the satisfaction of patients by reducing unnecessary costs and the degree of severity that occurs from medication errors.

The study has a few limitations. This analysis did not assess the medication errors that occurred regarding patients during hospitalization. The overall impact of clinical pharmacist interventions on healthcare cost was also not calculated. Additionally, the author was unable to evaluate readmission due to time constraints.

## 5. Conclusions

This 6-month retrospective study shows that prescription review and clinical pharmacist interventions influenced clinical outcomes, resulting in fewer days spent in the hospital. Clinical pharmacists played a critical role in optimizing drug therapy by preventing drug-related issues. The presence of a clinical pharmacist in the ICU ward has the potential to lower drug costs for both the patient and the hospital. The therapeutic optimization and prevention of pharmaceutical errors by pharmacist analysis and validation of prescriptions allow for significant public health financial savings, making this service extremely efficient. More research is needed to perform a thorough cost-benefit analysis. It is equally necessary for policymakers to consider this element to attain hospital service excellence.

## Figures and Tables

**Table 1:** Type of Clinical intervention by pharmacists.

	*Count*	*Percentage*
***Type of intervention***		
*Safety*	153	38
*Dosing*	20	4.9
*Indication*	185	45.7
*Miscellaneous*	46	11.4
*Total intervention*	404	100
***Interventions related to indication***		
*Drug with no indications*	38	18.5
*Add drug*	162	79
*Duplication*	5	2.4
*Total*	205	100.0
***Interventions related to safety***		
*TDM*	7	5.2
*Renal dose adjustment*	50	37.6
*Drug discontinuation due to long duration*	74	55.6
*Drug interaction*	2	1.50
*Total*	133	100.0
***Interventions related to dosing***		
*Dosing*	17	85
*Frequency*	3	15
*Total*	20	100.0
***Miscellaneous***		
*Provide information for nurse*	16	35
*Consultation*	30	65
*Total*	46	100.0
***Interventions related to cost saving***		
*Renal dose adjustment*	50	12.4
*Drug discontinuation due to long duration*	74	18.3
*Drug with no indications*	38	9.4
*Duplication*	5	1.2
*Drug interaction*	2	0.5
*Total*	169	41.6
***Intervention acceptance by physician***		
*Accepted*	370	91.5
*Not accepted*	36	8.5
Total	404	100.0

## Data Availability

The data that support the findings of this study are available from the corresponding author upon reasonable request.
